# Ingredients from Climate Resilient Crops to Enhance the Nutritional Quality of Gluten-Free Bread

**DOI:** 10.3390/foods11111628

**Published:** 2022-05-31

**Authors:** Megan Roozen, Luca Serventi

**Affiliations:** Department of Wine, Food and Molecular Biosciences, Faculty of Agriculture and Life Sciences, Lincoln University, Lincoln 7647, New Zealand; megan.roozen@lincolnuni.ac.nz

**Keywords:** gluten-free, sourdough fermentation, aquafaba, resilient grains, tapioca, brown rice, *Propionibacterium freudenreichii*, bread quality, nutrition

## Abstract

One percent of the global population requires a gluten-free diet. With concurrent global warming and population growth, it is increasingly necessary to optimize the use of ingredients from resilient crops, such as tapioca. Tapioca flour is used in low proportions in bread due to its lack of gluten. Sourdough fermentation can enhance the nutritional value of bread but also causes a sour taste. *Propionibacterium freudenreichii* subsp. *globosum* can reduce food acidity while synthesizing several nutrients, such as vitamin B12. Aquafaba is a known hydrocolloid and prebiotic. Therefore, the objective of this study was to test the sourdough fermentation of a composite bread based on tapioca and brown rice flour, cultured with *Lactobacillus lactis* and *Propionibacterium freudenreichii* subsp. *globosum* enriched in aquafaba. The bread quality was measured instrumentally (hardness, volume, moisture content) and with a semi-trained sensory panel (focus group). The co-fermentation of the *Lactobacillus lactis* and *Propionibacterium freudenreichii* subsp. *Globosum* produced palatable bread, improving the appearance, taste, and texture in comparison to the yeast-leavened recipe. This co-fermentation also enabled shorter production times, reducing it from 1 h to 30 min. The addition of the aquafaba further improved the bread appearance, texture, and volume, although a bitter tasting crust was reported. The co-fermentation of the tapioca–brown rice composite flour with *Lactobacillus lactis* and *Propionibacterium freudenreichii* subsp. *globosum* produced acceptable bread, which could provide a climate-resilient solution to food sustainability. The aquafaba addition further enhanced such improvements and the baking performance, offering sustainability in terms of nutrition, sensory quality, and price.

## 1. Introduction

With the approach of global warming concurrent with population growth, nutritious staple foods must be developed using sustainable ingredients from resilient crops, which can thrive within an altered climate. Cassava (*Manihot esculenta*) is a tropical crop that can tolerate a wide range of climatic conditions, including drought, as well as having the ability to grow in areas with low soil fertility, where many other crops are unable to grow [1]. Cassava yields the highest amount of carbohydrate per cultivation area of any crop [2], with low investment and irrigation requirements. It also provides minerals and vitamins, though it is low in protein [1]. It has an advantage for food security, being able to be stored underground for harvest when required [1].

Tapioca flour is made from the starch of cassava roots [3]. The use of tapioca flour in baked goods presents functionality issues, depending on dosage. For example, while 10% of tapioca starch improved the specific volume and increased the springiness of bread made with rice and semolina [3], and 10–20% improved air cell homogeneity in rice bread [4], more than 20% of fermented cassava flour reduced the quality of bread made with wheat, reducing crumb pore size and volume and creating a chewy texture [5]. When blended with rice starch, tapioca flour resulted in bread with large holes, attributed to the small, agglomerated granules [6]. The use of tapioca can also reduce baking volume in comparison with cereal flours due to low lipid levels [6]. Lipids provide stabilization by forming films at gas–liquid interfaces, increasing gas inclusion [6]. Consequently, tapioca is often used in small quantities, but sourdough fermentation may enable bread making with higher proportions of tapioca, as exopolysaccharides produced by bacteria may improve the structure and texture [7].

Tapioca is inherently gluten-free. Gluten-free bread is an essential staple food for an increasing population worldwide, yet it is nutritionally, texturally, and organoleptically inferior to gluten-containing bread [8]. Sourdough fermentation can improve the nutritional, textural, and sensory quality of bread [7], so this technology may be especially useful for gluten-free baked goods. Sourdough bread is a blend of flours and water fermented with lactic acid bacteria and yeast. The resulting metabolism produces bioactive compounds such as exopolysaccharides and enzymes that stabilize the protein scaffolding as well as the moisture dynamics, in addition to enhanced nutrient bioavailability [9]. The nutritional benefits of sourdough include reduced antinutrient levels, increased protein digestibility, and reduced allergenicity [10] from the synthesis of enzymes and the activity of organic acids, and exopolysaccharides [7]. Proteolytic activity within the sourdough can reduce phytic acid, consequently increasing the bioavailability of minerals and amino acids [7]. Sourdough bread is rich in volatile compounds, flavonoids [11], phenols from enzymatic hydrolysis, and soluble dietary fiber [12]. The glycemic index, often higher in gluten-free baked goods, is reduced in sourdough, as starch digestibility is lowered [7]. The exopolysaccharides created improve the viscoelasticity, volume, and hardness of bread [7], improving the texture. Furthermore, sourdough bread is softer [13] and has a stronger flavor and aroma than yeast bread due to higher levels of volatiles, flavonoids [10], and acidity.

The selection of the bacteria used to ferment sourdough may provide additional nutritional enhancement to gluten-free bread. Specifically, the *globosum* strain of *Propionibacterium freudenreichii* can produce active vitamin B12 (cyanocobalamin) [14], conjugated linoleic acid [15], and folate [16]. *Propionibacterium freudenreichii* also has the potential to enhance the sensory quality of gluten-free bread due to the acid and volatile aroma compound levels and the increased carbon dioxide production for leavening [14]. Propionic acid bacteria have been traditionally important for food preservation, as well as for aroma and flavor attributes [17]. Propionic acid produced by *Propionibacterium freudenreichii* fermentation is less acidic in flavor than lactic acid [18]. Therefore, it will be beneficial to ascertain its efficacy for in situ use in sourdough.

Chickpea cooking water (aquafaba) is also not normally used within sourdough. However, it may contribute structural and gas retention advantages, allowing higher-than-normal proportions of tapioca to be included in the bread. It expresses strong gelling and foaming ability from water-soluble polysaccharides [19]. As a by-product that is usually discarded, it may also supply waste reduction and cost advantages. It consists of about 5/100 g of solids, of which 1–1.5 g is protein and the rest is soluble carbohydrates, saponins, and minerals [19].

Consequently, our research focused on assessing the effects of co-fermenting tapioca flour and brown rice flour with *Propionibacterium freudenreichii* subsp. *globosum* and *Lactobacillus* lactis on bread quality, in terms of the moisture content, hardness, and sensory profile.

## 2. Materials and Methods

### 2.1. Materials

Numerous variables were evaluated: lactic fermentation, propionic fermentation, proofing time, and addition of aquafaba. A yeast-based bread was developed as control using tapioca flour (Pams, Auckland, New Zealand), brown rice flour (Pams, Auckland, New Zealand), canola oil (Pams, Auckland, New Zealand), salt (Pams, Auckland, New Zealand), yeast (Edmonds Active All Purpose, Christchurch, New Zealand), and sugar (Chelsea, Auckland, New Zealand). Sourdough was made with alternative bacterial starters, including *Lactobacillus lactis* (L), *Propionibacterium freudenreichii* subsp. *globosum* (strain PS-1) freeze-dried powder 0.5 units (The Urban Cheese Co., West Melton, New Zealand) (P). Co-fermentation was obtained with *Lactobacillus lactis* (freeze-dried powder, 2.5 units, Mad Millie, Auckland, New Zealand) and *Propionibacterium freudenreichii* without (CF) and with (CF + A) the addition of aquafaba. The recipes for these recipes are shown in Table 1. These were then mixed, in equal proportions, with the yeast dough. A 50:50 blend of tapioca flour (Pams, Auckland, New Zealand) and brown rice flour (Pams, Auckland, New Zealand), was hydrated with 160 mL of water and inoculated with bacterial starters, then incubated at 37 °C for 24 h. Later, the sourdough was mixed with 50 g of tapioca flour, 50 g of brown rice flour, 16 mL of canola oil, 6 g of salt, 8 g of yeast, and 8 g of sugar. Aquafaba was obtained from canned chickpeas (Chantal Organics, Napier, New Zealand) by draining them of their cooking water. The amount of tapioca flour was selected to ascertain the potential of sourdough fermentation to enable palatable bread to be created with this high proportion of tapioca. Brown rice flour was selected for the remaining flour proportion, to complement the tapioca by providing higher amounts of vitamins, minerals, fiber, and protein [20,21] and for maintaining a higher fermentation pH compared to other grains [22]. Brown rice is also suitable because it contains riboflavin (0.98 µg/g) that is required for vitamin B12 production [22]. Proximate composition of the commercial flours was the following: tapioca flour (0.1 g protein, 87.5 g carbohydrates), brown rice flour (7.7 g protein, 72.8 carbohydrates, of which 3.9 g dietary fiber, 2.6 g lipids).

### 2.2. Breadmaking

Sourdough was made with the ingredients in Table 1. Cultures were activated in water at room temperature (20 °C) for 3 min before combining with all of the other ingredients. The batter was mixed on speed 1 for 1 min in a bencthop mixer (Delta Food Equipment Mixer 500A, Oakville, Ontario, Canada), then medium speed for 4 min. The sourdough was then incubated in closed jars for 24 h at 30 °C (Sanyo incubator MIR-153, Kyoto, Japan).

After the sourdough incubation, yeast dough was prepared. Sugar and yeast were first combined for a minimum of 5 min in 20 mL of warm water (35 °C) and then added to the remaining ingredients. Sourdough was combined with yeast dough in equal proportions and mixed at speed 1 for 1 min, then speed 4 for 4 min. Resulting dough was then proofed covered in loaf tins (120 g/tin, dimensions 10 cm long, 6.5 cm wide, and 3 cm deep) at 30 °C (Sanyo incubator MIR-153, Kyoto, Japan). All recipes (with 3 loaves made per recipe) were made once with 30 min of proofing and again with 1 h of proofing. The 3 loaves from each recipe were baked together for 20 min at 180 °C (Moffat oven E32M, New Zealand).

### 2.3. Instrumental Analysis

After cooling for 20 min, baked bread was stored at room temperature in plastic bags for 24 h prior to instrumental analysis. Bread was weighed and volume was calculated by rapeseed displacement (AACC method 10-05) [23]. Specific volume was calculated as loaf volume/weight. Moisture content was determined by oven drying [24]. Briefly, a crumb sample from each baked loaf was heated at 105 °C for 5 h, and moisture content was calculated via the equation: (initial weight − final weight)/initial weight × 100. Crumb cubes of 25 mm thickness were prepared for texture analysis from 3 loaves of each sourdough recipe. A total of 5 crumb cubes were prepared per loaf, resulting in an average of 15 cubes tested per recipe. These crumb samples were tested with a Texture Analyser (TA.XT Plus, Stable Micro Systems. Godalmig, Surrey, UK). Compression was performed using a P/25 (25 mm diameter) aluminium probe, pre-test speed 1.0 mm/s, test speed 2.0 mm/s, post-test speed 10.0 mm/s, autotrigger type of 5 g and a 50 kg load cell (500 N).

### 2.4. Sensory Analysis

#### 2.4.1. Sensory Focus Group

The sensory focus group was composed of 9 student at Lincoln University. They were a multicultural group of females between the ages of 20 to 50, ‘semi-trained’ with existing food science knowledge, experience of sensory analysis, and the focus group training provided. This group was chosen as representative of average consumers (of gluten and non-gluten foods), with knowledge of food science to enable provision of detailed information on the technologies tested.

#### 2.4.2. Focus Group Training

Samples of 3 commercial bread products were sequentially provided to offer a wide range of attributes that could cover the samples tested. These commercial products included wholegrain yeast wheat bread (Sunny Crust Wholemeal, Auckland, New Zealand), gluten-free yeast bread (Vogel’s White, Auckland, New Zealand), and gluten-free sourdough bread (Gluten Freedom Sweet Potato Sourdough, Christchurch, New Zealand); ingredients in Appendix A. Sensory descriptors for appearance, aroma, taste, and texture for these breads were generated and agreed on by the panel, comparing attributes of regular wheatmeal bread with gluten-free bread and gluten-free sourdough bread. This was to provide sensory characteristic references and a collective descriptive vocabulary for the experimental gluten-free sourdough breads being assessed. A similar approach was used in other descriptive studies [25,26].

#### 2.4.3. Sample Analysis

Bread samples (approximately 30 mm cubed) were stored at −20 °C for ≤13 days, and thawed at room temperature (20 °C) for 3 h before sensory analysis. Water and plain crackers were provided for palate cleansing when required. Descriptors of each sample were collected, as well as the number of panel participants that agreed with each descriptor proposed.

### 2.5. Statistical Analysis

Instrumental data, including specific volume, moisture content, and hardness, were statistically analyzed via One-Way Analysis of Variance (ANOVA) with Tukey testing with Minitab20, assuming equal variances and a confidence interval of 95% (α = 0.05).

## 3. Results

### 3.1. Instrumental Analysis

#### 3.1.1. Long Fermentation (1 H)

**Figure 1:**
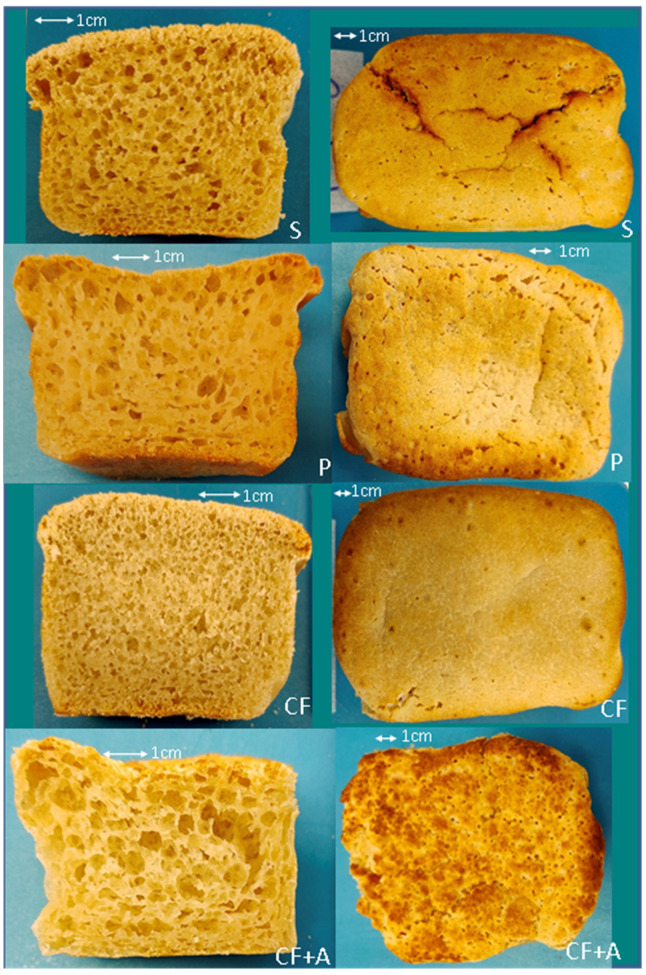
Crumb cross-section (**left**) and crust top view (**right**) of sourdough bread, fermented for 1 h, with alternative starters including spontaneous fermentation (S), *Propionibacterium freudenreichii* subsp. *globosum* (P), co-fermented with *Propionibacterium freudenreichii* subsp. *globosum* and *Lactobacillus lactis* (CF), and co-fermented with *Propionibacterium freudenreichii* subsp. *globosum* and *Lactobacillus lactis* with aquafaba included in the formulation (CF + A).

The instrumental quality of the sourdough formulations is shown in Table 2. After proofing, the *Lactobacillus lactis* (L) sourdough, having over-risen, collapsed and was unable to be analyzed. The co-fermented sourdough with aquafaba (CF + A) was also over-risen and consequently uneven (Figure 1). All the samples were significantly different from each other in hardness (*p* = 0.000) with the co-fermented sourdough (CF) being substantially harder than the other three groups. The *Propionibacterium freudenreichii* sourdough (P) was appealingly soft, while the co-fermented with aquafaba (CF + A) was too soft and difficult to slice. Only the co-fermented sourdough (CF) approached the hardness of the yeast-only bread (not sourdough) reference, so sourdough fermentation was able to significantly soften the gluten-free bread (which tends to be too hard) made with 50% tapioca flour.

The moisture content did not differ significantly across groups, while all the groups were significantly different in mean specific volume. As hypothesized, the sourdough co-fermented with aquafaba (CF + A) had a significantly higher specific volume than all the other groups, and the lowest specific volume was the co-fermented sourdough (CF), which was also the hardest. The crust appearance (Figure 1) was best for the *Propionibacterium freudenreichii* (P) and the co-fermented (CF) loaves, while the spontaneous fermentation sourdough (S) had obvious cracking of the crust, and the co-fermented with aquafaba (CF + A) crumb was irregular with many small holes. The crumb (Figure 1) was also the best for *Propionibacterium freudenreichii* (P), with the spontaneous (S) second, with fairly even pores, while the co-fermented sourdough (CF) had too little aeration, and the co-fermented with aquafaba (CF + A) had large holes in places. The co-fermented with aquafaba (CF + A) had more crust browning than all the other samples.

#### 3.1.2. Short Fermentation (30 Min)

**Figure 2:**
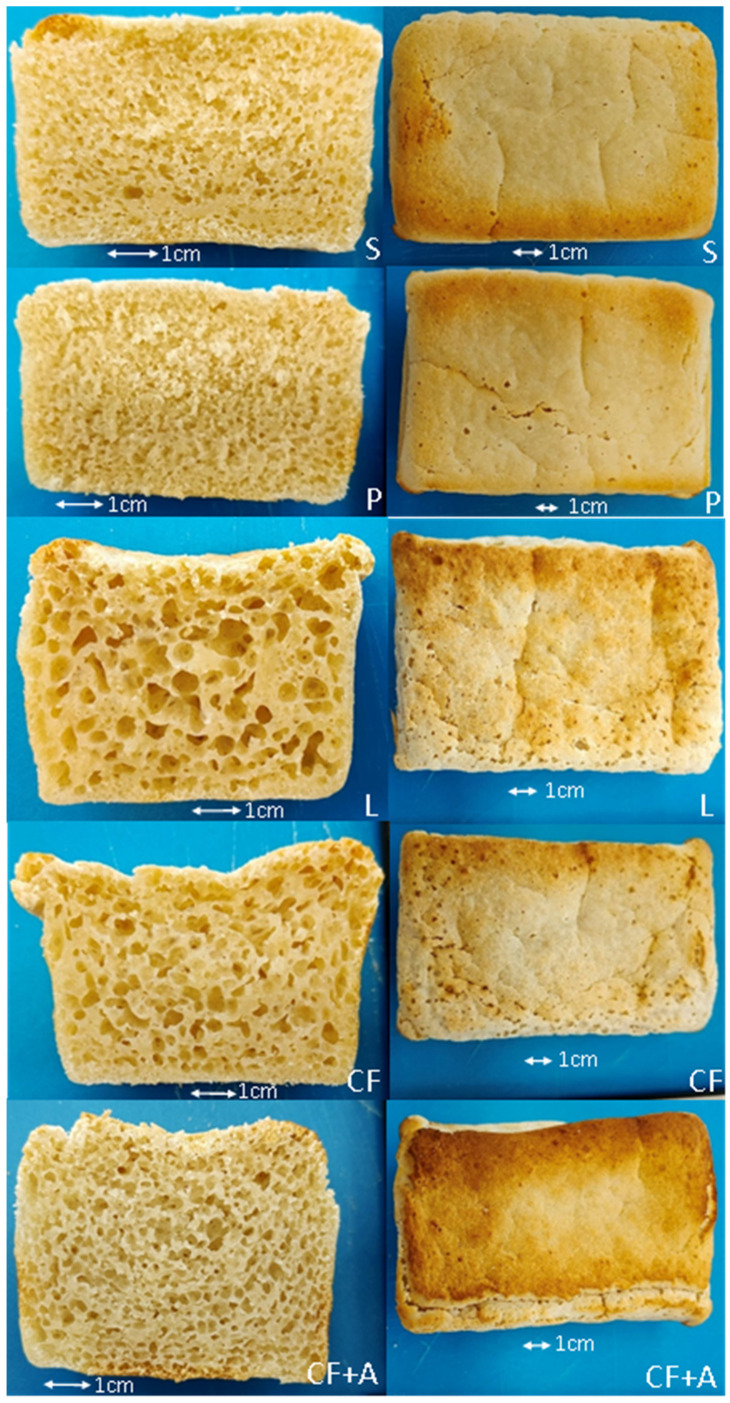
Crumb cross-section (**left**) and crust top view (**right**) of sourdough bread, fermented for 30 min, with alternative starters including: spontaneous fermentation (S), *Propionibacterium freudenreichii* subsp. *globosum* (P), *Lactobacillus lactis* (L), co-fermented with *Propionibacterium freudenreichii* subsp. *globosum* and *Lactobacillus lactis* (CF), and co-fermented with *Propionibacterium freudenreichii* subsp. *globosum* and *Lactobacillus lactis* with aquafaba included in the formulation (CF + A).

The instrumental quality of the sourdough formulations proofed for only 30 min are shown in Table 3. The *Lactobacillus lactis* (L) sourdough had optimal rising (Figure 2). The overall mean hardness across all the groups was significantly higher for 30 min of proofing compared to 1 h (630 and 910 g, respectively). This was advantageous for the co-fermented with aquafaba sourdough (CF + A) because it had been too soft with the 1 h proofing. However, the sourdough co-fermented with aquafaba (CF + A) was still significantly softer than all the other groups, but with a desirable level of softness this time (576 g) (Table 3). The *Propionibacterium freudenreichii* (P), the hardest of all the groups, was substantially harder with the shorter proofing time. The *Lactobacillus lactis* (L) was the second softest group, and the co-fermented (CF) bread was acceptably soft.

The moisture content did not vary with formulation, but the co-fermented with aquafaba (CF + A) and *Lactobacillus lactis* (L) had a significantly higher specific volume than the *Propionibacterium freudenreichii* (P), which had the lowest specific volume (Table 3). In terms of appearance, the co-fermented with aquafaba (CF + A) improved both the crust color with browning and the crumb structure with even pores (Figure 2). The *Lactobacillus lactis* (L) and the co-fermented (CF) had some over-large holes in the crumb, with holes also worsening the crust appearance. The *Propionibacterium freudenreichii* (P) crumb was dense in places.

### 3.2. Descriptive Sensory Analysis

The consensus descriptors (from the focus panel) of the sensory characteristics, appearance, aroma, taste, and texture, of the commercial breads used for reference training are shown in Table 4. The terms that were selected for the experimental sample breads are shown in bold. Table 5 shows the descriptors used for the sample breads from each recipe, including the reference consensus descriptors when selected and new descriptors that were not used for the commercial breads. The number of participants that agreed to each descriptor was recorded.

## 4. Discussion

### 4.1. The Effect of Sourdough Fermentation on the Quality of the Tapioca–Brown Rice Bread

All the sourdough bread samples tested were softer than the yeast bread (1394 and 1490 g for CF and yeast, respectively). The previous literature has highlighted the soft texture in sourdough bread, proportional to fermentation time [9,27]. The sourdough fermentation improved the tapioca bread appearance, with a stronger color, less crumb holes, and less cracking of the crust. Crust cracking can occur when connections between particles within the dough matrix are weak, so gas is able to move through the dough and crack the crust on escape [28]. It may be that the exopolysaccharides produced by bacteria during the sourdough fermentation strengthen the dough structure, improving air retention [9].

All the sourdough bread recipes contained high levels of moisture content (from 42 to 50%), with no significant difference across recipes. This is expected because tapioca as a tuber starch retains a higher level of moisture than cereal starches [6]. A previous study [20] found that 30% tapioca starch prevented water from evaporating in rice and tapioca bread. Lower specific volumes are also expected when using tapioca, which is known to have low levels of gas incorporation in dough [20], and our results confirm this. The sourdough effect on bread volume is however contrasting. While traditional cultures based on *Lactobacillus* spp. may reduce volume [27], the *Propionibacterium freudenreichii* were shown to enhance it [28]. The differences are ascribed to the production of lactic acid in the former case and exopolysaccharides in the latter [9]. However, the *Propionibacterium freudenreichii* did not enhance the specific volume when used in the tapioca sourdough.

### 4.2. The Effect of Alternative Starter Cultures on the Quality of the Tapioca–Brown Rice Bread

The traditional sourdough bread was spontaneously fermented. This can lead to less control over fermentation characteristics and, consequently, inconsistent bread quality [7,9,29]. The spontaneous fermentation (S) resulted in tapioca bread with an even crumb appearance and acceptable hardness (747–943 g) and specific volume, but with the most surface cracking and an unappealing texture. When starter cultures were used for sourdough fermentation, lactic acid bacteria were the usual choice [7,9]. The fermentation of dough with this bacterium was associated with a soft texture due to an enhanced structure, consequence of the exopolysaccharides produced [9,28]. Our results reflected this, as the *Lactobacillus lactis* (L) loaves were the second softest in the instrumental analysis (750 g), with the second-highest specific volume (2.31 cm^3^/g). However, the *Lactobacillus lactis* (L) was characterised with an uneven crumb with some medium-sized pores and holes in the crust. According to the sensory focus panel, it formed a springy, crumpet-like texture with a bad aftertaste.

*Propionibacterium freudenreichii* is less acidic than lactic acid bacteria [18], so it was expected to produce bread with a less acidic flavor and aroma. This proved true with our sensory focus panel reporting a less acidic flavor and aroma in the *Propionibacterium freudenreichii* (P) sourdough, but instead of the acidity, all the participants experienced a bitter aftertaste. Such changes are attributed to the lower acidity and higher bitterness of the propionic acid in comparison to the lactic acid. The panel also found the *Propionibacterium freudenreichii* bread to be texturally unappealing, and this matched the instrumental data where hardness was significantly higher, and specific volume significantly lower, than the other breads tested. However, this was improved by a longer fermentation time (1 h proofing), and after this treatment, *Propionibacterium* loaves were one of the most promising formulations made, with a desirable softness (256 g) and an improved crust and crumb appearance.

Loaves that were co-fermented with *Lactobacillus lactis* and *Propionibacterium freudenreichii* subsp. *globosum* (CF) had the most positive sensory testing responses from the focus panel, for both taste and texture. This formulation was the most promising of all the loaves with the shorter proofing time, making a promising tapioca sourdough bread with a notable reduction in production time. This could offset the greater comparative cost of gluten-free bread ingredients and production. While co-fermented (CF) loaves had a significantly lower mean specific volume than other formulations, they had an acceptable hardness (853 g) and a reasonable appearance with evenly distributed pores in the crumb.

### 4.3. The Effect of the Addition of Aquafaba on the Quality of the Tapioca–Brown Rice Bread

The tapioca sourdough loaves that were co-fermented with *Lactobacillus lactis* and *Propionibacterium freudenreichii* subsp. *globosum* with the addition of aquafaba (CF + A) were also best with the shorter proofing time, providing the advantage of a reduced production time. The air retention was likely enhanced by the aquafaba because it is a strong foaming agent, with capacity up to 58–548% due to the soluble protein and saponins [30,31]. The structure-forming hydrocolloid capacity of aquafaba [31] is potentially the reason these loaves had the highest specific volume (2.39 cm^3^/g) and were significantly softer than the other formulations (576 g). Bird and others [32] also found that aquafaba reduced the crumb hardness of the gluten-free yeast bread (made with equivalent amounts of rice and corn flours), but with greater resulting hardness (2975 g) than the values found here for gluten-free sourdough bread. The dough made with aquafaba was more viscous than the other formulations, and viscosity is important for gas retention [33]. Furthermore, the aquafaba exhibited strong prebiotic activity which causes the exponential growth of bacteria (such as *Lactobacillus*), ascribed to the content of oligosaccharides, such as raffinose, stachyose, and verbascose, as well as free amino acids and minerals [30,31].

The sourdough bread containing aquafaba (CF + A) showed the best appearance, with the most consistent crumb (with many small, evenly distributed pores), and the crust was even with the best browning observed. This is likely to be from the Maillard’s reaction occurring due to amino acids and sugars not normally present in gluten-free bread, available from the aquafaba [32]. The sensory testing reported the appearance to be uniform and attractive. The texture was also better than most other formulations (though still mentioned to be sticky and chewy), but there was a bitter tasting crust. Further research could ascertain if the bitterness tasted in the co-fermented with aquafaba (CF + A) bread is attributable to the *Propionibacterium freudenreichii* or to the aquafaba.

## 5. Conclusions

Sourdough co-fermentation with *Propionibacterium freudenreichii* and *Lactobacillus lactis* enabled palatable bread to be made using a high proportion of tapioca flour (50% of the flour blend). Co-fermentation with these bacteria improved the bread appearance, taste, and texture and also enabled shorter production times, with proofing time reduced by 50%. The addition of the aquafaba further improved the bread appearance, texture, and volume, but a bitter tasting crust was reported. Further research could include the effect of sourdough viscosity, fermentation time, and light levels available during incubation on vitamin B12 production in sourdough. An assessment of vitamin B12 availability in sourdough would confirm the merit of in situ natural fortification.

## Figures and Tables

**Table 1:** Sourdough recipes with alternative starters including spontaneous fermentation (S), *Propionibacterium freudenreichii* subsp. *globosum* (P), *Lactobacillus lactis* (L), co-fermented with *Propionibacterium freudenreichii* subsp. *globosum* and *Lactobacillus lactis* (CF), and co-fermented with *Propionibacterium freudenreichii* subsp. *globosum* and *Lactobacillus lactis* with aquafaba included in the formulation (CF + A).

Ingredients (g)	S	P	L	CF	CF + A
Tapioca flour	100	100	100	100	100
Brown rice flour	100	100	100	100	100
Water	160	160	160	160	20
*Propionibacterium freudenreichii* subsp. *globosum*	0	0.3	0	0.3	0.3
*Lactobacillus lactis*	0	0	1.0	1.0	1.0
Aquafaba	0	0	0	0	148

**Table 2:** Instrumental evaluation of bread fermented for 1 h. Different superscripts refer to statistically significant values.

Bread Formulation	Hardness (g)	Specific Volume (cm^3^/g)	Moisture Content (%)
S	747 ± 116 ^B^	2.50 ± 0.12 ^B^	44.7 ± 2.0 ^A^
L	Nonmeasurable	Nonmeasurable	Nonmeasurable
P	256 ± 37 ^C^	2.10 ± 0.11 ^C^	45.2 ± 1.3 ^A^
CF	1394 ± 190 ^A^	1.82 ± 0.10 ^D^	44.5 ± 3.1 ^A^
CF + A	38 ± 38 ^D^	2.86 ± 0.10 ^A^	42.0 ± 3.28 ^A^
Yeast only (reference)	1490 ± 235	Not measured	Not measured

**Table 3:** Instrumental evaluation of bread fermented for 30 min. Different superscripts refer to statistically significant values.

Bread Formulation	Hardness (g)	Specific Volume (cm^3^/g)	Moisture Content (%)
S	943 ± 90 ^B^	1.86 ± 0.24 ^AB^	46.0 ± 2.2 ^A^
L	750 ± 128 ^C^	2.31 ± 0.33 ^A^	46.7 ± 0.8 ^A^
P	1461 ± 191 ^A^	1.56 ± 0.16 ^B^	46.8 ± 1.5 ^A^
CF	853 ± 127 ^BC^	2.07 ± 0.06 ^AB^	47.0 ± 1.5 ^A^
CF + A	577 ± 93 ^D^	2.39 ± 0.29 ^A^	50.0 ± 2.8 ^A^

**Table 4:** Commercial bread sensory analysis descriptors selected by the focus group panel.

**Sensory** **Characteristic**	**Wholemeal**	**Gluten-Free** **White**	**Gluten-Free** **Sweet Potato** **Sourdough**
**Appearance**	Spotty Grainy	**Uniform** Processed	Brown **Attractive** Holey (air bubbles)
**Aroma**	**Bready** Familiar Wheaty	**Neutral**	**Sour** Yeasty Bready **Fermented**
**Taste**	**Pleasant** **Bready** Familiar **Sweet** **Yeasty**	Stale Bland Lingering	Sweet **Sour** **Vinegar**
**Texture**	Soft Fluffy Grainy	**Dry** Mushy Gritty Stale Kitchen Sponge **Sticky**	Dry Hard **Uniform** Crumbly

**Table 5:** Sensory analysis descriptors selected for the sourdough samples.

Sensory Characteristic	S	L	P	CF	CF + A
**Appearance**	**Dense 9** **Grey 6** **Undercooked 5**	**Pale 8** **Tan Crust 5** Attractive 4 Yellower 4 Rustic 1	**Dense 9** **Pale 9** Undercooked 1	**Rustic 9** **Holey Crumb 9** **Pale 8** Flaky Crust 2	**Uniform 9** **Attractive 7** **Bready 6** Less Crumbly 1 Less Holes 1
**Aroma**	**Sour 7** **Beer Like 6**	**Sour 8** **Yeasty 5**	**Fermented 7** Sour Fruit 1 Kombucha 1 Unpleasant 1	**Sour 9** **Apple Cider 5** Acetic 4 Vinegar 3	**Sour 9** Roast Potato 1
**Taste**	**Neutral 9** Bitter 4 Tasteless 3 Salty 1	**Sour 8** **Acidic Aftertaste 6** Sweet 3 Pleasant 3	**Bitter Aftertaste 9** Less Sour 2	**Salty 6** Too Yeasty 2 A Little Bitter 2	**Bitter Crust 8** **Sour 6** Baking Soda 1 Bitter Aftertaste 1
**Texture**	**Chewy 9** **Dense 9** **Chalky 6** **Dry 5**	**Crumpet 9** **Springy 9** **Sticky 7**	**Heavy 9** **Chewy 9** **Gluey 9** **Sticky 9** **Dense 7**	**Doughy 9** **Chewy 9** **Sticky 8**	**Sticky 9** **Gluey 6** **Chewy 6**

## Data Availability

No new data were created or analyzed in this study. Data sharing is not applicable to this article.

## Appendix A. Ingredients of Commercial Breads Used for Focus Group Training and Descriptor Generation

**Ingredients**		
Sunny Crust Wholemeal	Vogel’s Gluten Free White	Gluten Freedom Sweet Potato Sourdough
Wheat flour (wholemeal and white) Water Wheat gluten Yeast Iodized salt Canola oil Soy flour Emulsifiers (471, 481) Acidity regulator (263) Vtamin (folic acid)	Water Modified tapioca starch (1442) Flour (rice, soy) Maize starch Canola oil Sugar gg white powder Yeast Iodized salt Psyllium Cultured dextrose White vinegar Stabilizers (412, 464)	Water Organic Sourdough (Brown Rice Flour, Water, Vegetable Gum (Guar Gum)) Modified Tapioca Starch (1442) Corn Starch Coconut Sugar Coconut Oil Kumara Powder (2.9%) (Sweet Potato) Psyllium Husk Polenta Yeast Iodized Salt Stabilizer (464) Vegetable Gum (Guar Gum) Emulsifier (Sunflower Lecithin)
